# Detection of a New Tert-Leucinate Synthetic Cannabinoid 5F-MDMB-PICA and Its Metabolites in Human Hair: Application to Authentic Cases

**DOI:** 10.3389/fchem.2020.610312

**Published:** 2020-11-26

**Authors:** Yan Shi, Liying Zhou, Le Li, Mengxi Liu, Huosheng Qiang, Min Shen, Baohua Shen, Hang Chen, Olaf H. Drummer, Wanhui Liu, Hejian Wu, Ping Xiang

**Affiliations:** ^1^Department of Forensic Toxicology, Shanghai Key Laboratory of Forensic Medicine, Shanghai Forensic Science Platform, Academy of Forensic Science, Shanghai, China; ^2^School of Pharmacy, Yantai University, Yantai, China; ^3^Department of Forensic Medicine, Faculty of Medicine, School of Public Health and Preventive Medicine, Nursing and Health Sciences, Monash University, Southbank, VIC, Australia

**Keywords:** synthetic cannabinoid, 5F-MDMB-PICA, metabolites, UHPLC-MS/MS, hair analysis

## Abstract

Methyl 2 -[ [ 1- (5- fluoropentyl) indole - 3- carbonyl] amino] -3, 3- dimethyl - butanoate (5F-MDMB-PICA) is a new synthetic cannabinoid characterized by valinate or tert-leucinate moieties. In recent years, 5F-MDMB-PICA has been abused in the form of “spice-like” herbal incenses or electronic cigarette oil. A UHPLC-MS/MS method was developed to detect 5F-MDMB-PICA and its metabolites in human hair. Approximately 20 mg of hair was weighed and pulverized with methanol below 4°C. After ultrasonication, centrifugation and filtration, 200 μL of supernatant was placed into an autosampler vial and analyzed on a Waters Acquity UPLC HSS T_3_ column (100 mm × 2.1 mm, 1.8 μm particle size) using an acetonitrile-20 mmol/L ammonium acetate (0.1% formic acid, 5% acetonitrile) gradient with a run time of 8 min. The limit of detection (LOD) ranged from 0.5 to 5 pg/mg, and the lower limit of quantitation (LLOQ) ranged from 1 to 5 pg/mg. The method was shown to be linear over a concentration range of 1–200 pg/mg. The linear correlation (*R*^2^) of the calibration curves for all analytes was >0.999. The accuracy varied from 95.4 to 107.4%, while the intra- and inter-day precision RSD values were 0.7–10.6% and 1.7–12.2%, respectively. Recoveries were within the range of 61.1–93.3%, and matrix effects were in the range of 19.1–102.6%. The validated method was successfully applied to the identification and quantification of 5F-MDMB-PICA and its metabolites in hair from authentic forensic cases.

## Introduction

Recently, new psychoactive substances (NPS) have surged at an alarming rate worldwide, which has severely impacted global health problems. Contrary to traditional drugs of abuse, the structures of NPS are diverse, and the pharmacokinetic and physiological properties of most of them have never been evaluated in controlled studies, which brings great challenges to their detection and interpretation (Smith et al., 2015; Risseeuw et al., 2017). Among NPS, synthetic cannabinoids (SCs) are still the fastest-growing class of NPS monitored by the Early Warning System (EWS) of the European Monitoring Centre for Drugs and Drug Addiction (EMCDDA) (2017). As of 2017, according to EMCDDA data, 179 synthetic cannabinoids were reported (Truver et al., 2020). Illegal drug dealers and secret laboratories use the structural diversity of SCs to evade analysis and detection and to circumvent prohibition by international legislation (Debruyne and Le Boisselier, 2015; Banister and Connor, 2018a,b). Due to the illegal abuse of a large number of synthetic cannabinoids in recent years, many serious poisoning and death cases have occurred worldwide (Hess et al., 2015; Weaver et al., 2015; Chinnadurai and Srijan, 2016). At present, 5F-MDMB-PICA is relatively common in China and is mainly sold on the Internet or in retail stores in the form of shredded tobacco, tobacco leaves or e-liquid.

Methyl 2-[[1-(5-fluoropentyl)indole-3-carbonyl]amino]-3,3-dimethyl-butanoate (5F-MDMB-PICA) belongs to the class of synthetic cannabinoids. This compound was synthesized and described for the first time by Banister et al. for the purpose of pharmacological research on novel SCs characterized by valinate or tert-leucinate moieties (Banister et al., 2016). The structure of 5F-MDMB-PICA is similar to that of 5F-MDMB-PINACA, but the indazole moiety is replaced by an indole group. 5F-MDMB-PICA was first detected in herbal incense packages by Risseeuw et al. (2017). In biological matrices, the parent structure of synthetic cannabinoids is often difficult to detect, so metabolic studies are needed to improve the detection of emerging synthetic cannabinoids. To date, only the detection of 5F-MDMB-PICA and its metabolites in urine have been reported (Mogler et al., 2018; Truver et al., 2020).

The detection and quantitative analysis of NPS in different biomatrices has become a great challenge for forensic toxicology research, mainly due to the need for sensitive, reliable and specific analytical techniques. Synthetic cannabinoids are usually monitored by analyzing the parent drug and its metabolites in blood and urine samples (Presley et al., 2016; Krotulski et al., 2019). Currently, hair is one of the key sample matrices for determining drug abuse in the field of forensic science (Salomone et al., 2014). Compared with that of biological matrices such as blood and urine, hair testing has the advantages of stable target compounds, a larger detection window for drug detection, and the ability to reflect drug usage for a longer period of time (Baumgartner et al., 1989). However, few studies have examined synthetic cannabinoids and their metabolites in human hair.

**Scheme 1:**
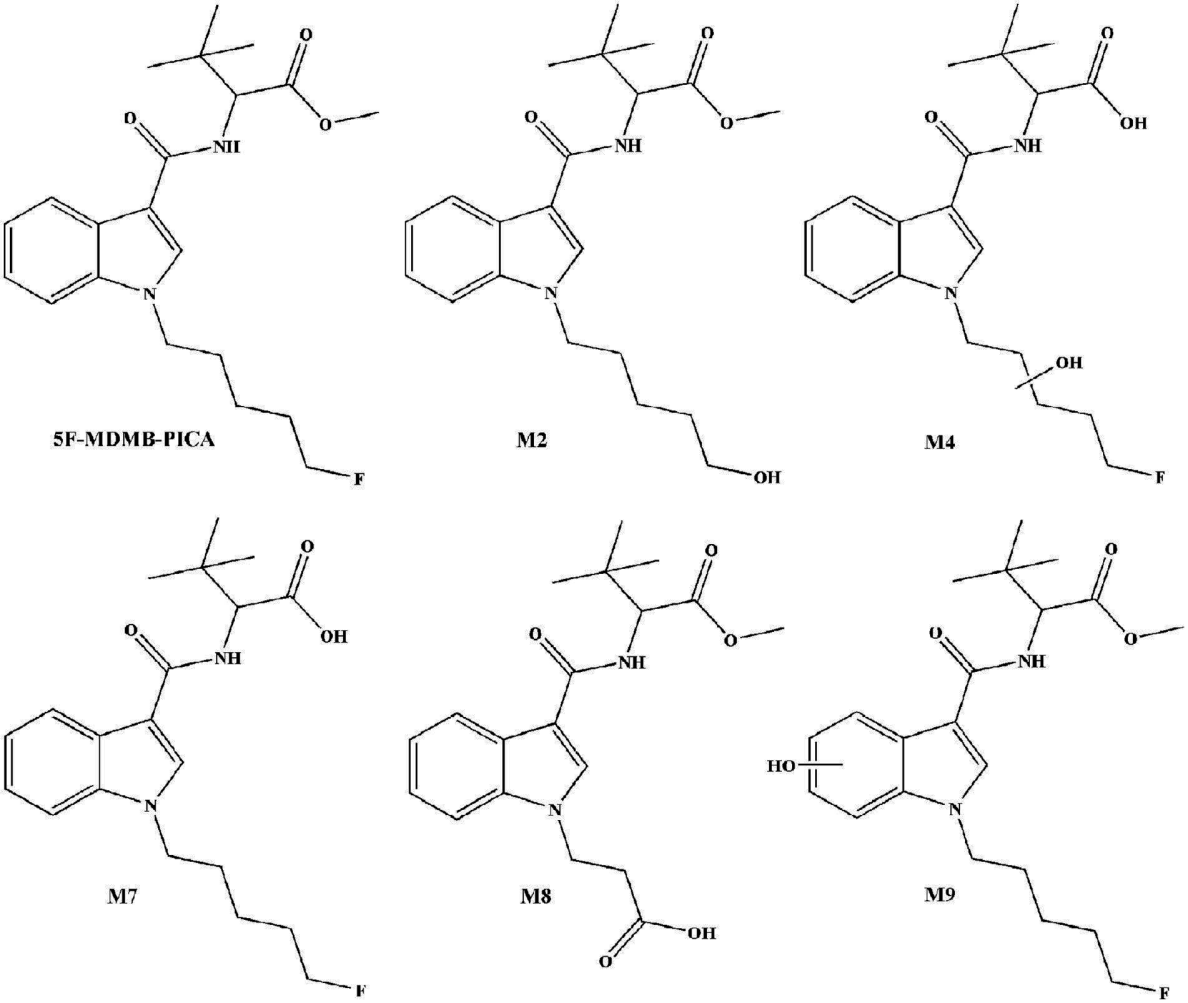
Chemical structures of 5F-MDMB-PICA and its metabolites used for method validation.

To our knowledge, analytical data on 5F-MDMB-PICA and its metabolites in hair have not been published. According to reference reports, the five metabolites M2, M4, M7, M8, and M9 in urine are the main metabolites of 5F-MDMB-PICA, so these five metabolites were selected as the identification metabolites of 5F-MDMB-PICA in human hair (Mogler et al., 2018). In this study, a validated method is presented for the quantitative determination of 5F-MDMB-PICA and its five metabolites in human hair by liquid chromatography-tandem mass spectrometry (Scheme 1). The method was successfully applied to authentic hair samples from real cases.

## Materials and Methods

### Reagents and Chemicals

A standard of 5F-MDMB-PICA was purchased from Cayman Chemical Company (Michigan, USA). Standards of metabolites M2, M4, M7, M8, and M9 were provided by Glpbio (California, USA). The deuterated internal standard (IS) JWH-018 4-hydroxypentyl metabolite-D5 was purchased from Cerilliant (Texas, USA). High-performance liquid chromatography-grade methanol and acetonitrile were obtained from Sigma-Aldrich (St. Louis, MO, USA). Analytical-grade isopropanol was provided by Shanghai Lingfeng Chemical Reagent Co. (Shanghai, China). Formic acid (≥98%) and ammonium acetate (≥98%) were obtained from Fluka (Buchs, Switzerland). Ultrapure water was prepared using a Millipore AFS-10 water purification system (Billerical, MA, USA).

### Hair Samples

Hair samples from healthy laboratory drug-free volunteers were used to prepare standard curves and quality control (QC) samples. Hair samples were obtained from suspected 5F-MDMB-PICA users. All individuals provided written informed consent.

### Preparation of Calibration Standards and Quality Control Samples

A 1 μg/mL stock solution containing 5F-MDMB-PICA and its metabolites was prepared with methanol. From this stock solution, working solutions with concentrations of 200, 50, 10, 5, 1, and 0.5 ng/mL were prepared with methanol. A 10 μg/mL IS stock solution was diluted with methanol to provide the IS working solution with a concentration of 100 ng/mL.

Calibration standards were prepared by spiking blank hair with the working solution to obtain final concentrations of 1, 2, 5, 10, 20, 50, 100, and 200 pg/mg for 5F-MDMB-PICA, M2, and M9; 2, 5, 10, 20, 50, 100, and 200 pg/mg for M4 and M7; and 5, 10, 20, 50, 100, and 200 pg/mg for M8. Quality control (QC) samples were prepared at four concentrations, namely, the LLOQ and low, medium and high concentrations. The QC concentrations of 5F-MDMB-PICA, M2 and M9 were 1, 10, 50, and 150 pg/mg; the QC concentrations of M4 and M7 were 2, 10, 50, and 150 pg/mg; and the QC concentrations of M8 were 5, 20, 50, and 200 pg/mg. All the standard stock solutions, working solutions and QC samples were stored at −20°C before use.

### Instrumentation

LC-MS/MS analysis was performed using an Acquity™ Ultra performance LC I-CLASS (Waters, USA) coupled with a Sciex 6500 Plus Q-trap™ quadrupole mass spectrometer (Sciex, Foster City, USA) with electrospray ionization (ESI). Data acquisition and processing were performed by MultiQuant 3.0.2.

Chromatographic separation was performed with a Waters Acquity UPLC HSS T_3_ column (100 mm × 2.1 mm, 1.8 μm particle size) using linear gradient elution. Mobile phase A (MPA) consisted of 0.1% formic acid, 20 mmol/L ammonium acetate and 5% acetonitrile. Mobile phase B (MPB) was acetonitrile. The gradient elution used was as follows: start at 0 min with 70% solvent A and 30% solvent B, held for 1 min; 1–2 min linear rate to 85% B, 2–6 min linear rate to 90% B; 6–7 min with 90% B, held for 1 min; 7–7.1 min return to initial conditions, 30% B; 7.1 8 min with 30% B, held for 0.9 min. was programmed as shown in Table 1. The flow rate was 0.3 mL/min, and the total run time was 8 min. The injection volume was 5 μL.

**Table 1:** MRM parameters and retention times for 5F-MDMB-PICA, its metabolites and the IS.

**Analytes**	**Formula**	**Precursor ion (*m/z*)**	**Product ion (*m/z*)**	**DP (eV)**	**CE (eV)**	**Rt (min)**
5F-MDMB-PICA	C_21_H_29_FN_2_O_3_	377.2	232.2^*^	50	25	3.35
			144.0	50	55	3.35
M2	C_21_H_29_FN_2_O_3_	375.3	230.0^*^	75	22	2.89
			144.0	75	48	2.89
M4	C_20_H_27_FN_2_O_4_	379.1	248.2^*^	60	18	2.44
			143.9	60	44	2.44
M7	C_20_H_27_FN_2_O_3_	363.1	232.0	55	23	2.85
			144.0^*^	55	51	2.85
M8	C_19_H_24_N_2_O_5_	361.1	216.0	40	20	2.69
			144.0^*^	40	55	2.68
M9	C_21_H_29_FN_2_O_3_	393.1	248.1^*^	60	21	2.88
			240.1	60	32	2.88
JWH-014 Hydroxypentyl metabolite-D5	C_24_H_18_D_5_NO_2_	363.3	155.0^*^	80	28	3.12
			127.0	80	60	3.12

* *quantifier ions; DP, declustering potential; CE, collision potential; Rt, retention time*.

The mass spectrometer was operated in multiple reaction monitoring (MRM) mode. The ESI source settings were as follows: source mode, positive; source temperature, 500°C; curtain gas (CUR, nitrogen), 18 psi; ion spray voltage (ISV), 5,500 V; collision cell exit potential (CXP), 10 V; entrance potential (EP), 10 V; collision activation dissociation (CAD) gas, low; ion source gas 1 (GS1), 40 psi; and ion source gas 2 (GS2), 35 psi. A summary of the MRM parameters and retention times is shown in Table 1.

### Sample Preparation

Hair samples were consecutively washed once with isopropanol and twice with water and dried at room temperature. The hair samples were then cut into 1–2 mm pieces with scissors for further study. Hair (20 mg) was weighed into 2 mL tubes. Mix the extraction solvent methanol solution with 1 μg/mL IS working solution to prepare 20 pg/mg IS extraction solution. Ceramic beads (1 mm) were added before the addition of the mixture solution of methanol solution and the IS (20 pg/mg). Hair samples were then homogenized using the BeadRuptor system (OMNI, USA). The settings for pulverization were as follows: temperature, below 4°C; speed, 6 m/s; time, 20 s; dwell time 40 s; and cycles, 10. The samples were then sonicated in an ice bath for 15 min and centrifuged for 5 min at 13,500 × g. Approximately 200 μL of the supernatant was removed and filtered through a 0.22 μm filter membrane (Sinopharm Chemical Reagent Co., Ltd., China). The filtrate was transferred into the autosampler vial, and 5 μL was injected into the LC-MS/MS system.

### Method Validation

Analytical method validation was carried out according to international guidelines (Peters et al., 2007; Scientific Working Group for Forensic Toxicology, 2013; Desharnais et al., 2014). Validation parameters, such as selectivity, limit of detection (LOD), lower limit of quantification (LLOQ), linearity, accuracy, precision, recovery, matrix effect and stability, were evaluated.

#### Selectivity

Selectivity was evaluated by analyzing 10 blank hair samples obtained from ten drug-free volunteers to check potential endogenous interferences from matrix components with the signals of analytes and the IS.

#### LOD and LLOQ

The LOD was defined by evaluating the signal/noise (S/N) ratio of three replicates of spiked blank hair samples at decreasing concentrations. A S/N of at least 3:1 was selected as the LOD, while the LLOQ was determined as the concentration having a S/N ≥ 10, and both required accuracy and precision values < ±20%.

#### Linearity, Accuracy, and Precision

Linearity was verified from six duplicates with concentrations of 1, 2, 5, 10, 20, 50, 100, and 200 pg/mg from the “in-house” certified drug-free hair. The deviation value of the LLOQ sample on the linearity does not exceed 20%, and the deviation value of other samples does not exceed 15%. Accuracy refers to “the difference between the value of the test result and the acceptable reference value.” It is usually expressed as the percentage deviation of the average of the test results from the acceptable reference value. Precision refers to “the closeness of agreement (degree of scatter) between a series of the test results obtained from multiple sampling of the same homogenous sample under the prescribed conditions,” and is usually expressed in terms of relative standard deviation (RSD). Accuracy and precision were assessed for hair samples by measuring six replicates at the LLOQ and QC samples. Intra-day precision and accuracy were determined by analyzing six replicates on a single day, while inter-day precision and accuracy were evaluated by analyzing six replicates prepared daily for 4 days. The maximum acceptable accuracy for QC samples does not exceed 85–115%, and for LLOQ samples it does not exceed 80–120%. The CV value of intra-day precision and inter-day precision is not more than ±15% for QC samples and not more than ±20% for LLOQ samples.

#### Recovery and Matrix Effect

The matrix effect and recovery experiments were designed according to the experimental scheme proposed by Matuszewski et al. (2003). Recovery and matrix effects were measured by analyzing QC samples with six replicates. The samples were divided into three groups: pre-extraction spiked samples (A), post-extraction spiked samples (B) and neat solution (C). The matrix effect was calculated by B/C, and recovery was calculated by A/C.

#### Dilution Integrity

Hair samples at concentrations of 1,000 and 2,000 pg/mg were prepared. Hair samples with concentrations of 1,000 pg/mg (*n* = 6) and 2,000 pg/mg (*n* = 6) were diluted 10 times and 100 times, respectively, after extraction. Dilution integrity was evaluated by determining the precision and accuracy.

#### Stability

Three QC samples at low, medium and high concentrations were used from six batches and stored in the autosampler at 4°C for 24, 48, and 72 h to investigate the stability of 5F-MDMB-PICA and its metabolites in extracts.

### Application to Real Cases

The validated method was applied to four authentic forensic cases. All hair samples came from suspected drug users. The hair samples were washed, and 1–6 cm sections of the hair samples were cut and divided into 1–3 and 4–6 cm sections. Each section of hair was cut into ~1–2 mm pieces for analysis of the presence of drug of abuse. Four suspicious users provided written informed consent.

## Results and Discussion

### Method Development

To obtain good chromatographic separation and symmetrical peak shape, the chromatographic conditions were optimized. After investigation, different gradient mobile phases were able to separate 5F-MDMB-PICA and its metabolites at a flow rate of 0.3 mL/min. The retention times of 5F-MDMB-PICA and its metabolites are shown in Table 1. The collision energy and declustering voltage were optimized to obtain suitable precursor and product ions.

There have been some reports in the literature on the choice of organic solvents to extract synthetic cannabinoids in hair (Hutter et al., 2012; Salomone et al., 2012, 2014; Gottardo et al., 2014), although methanol is the most common solvent for extracting drugs from hair. To optimize the extraction solvent of 5F-MDMB-PICA and its metabolites, methanol, an EM solution (a mixture of methanol, acetonitrile, and 2 mM ammonium formate) and n-hexane:ethyl acetate (9:1, v/v) were used as extraction solvents to evaluate their extraction recovery. The result shows the extraction recoveries of 5F-MDMB-PICA and its metabolites using these different extraction solvents. The extraction recoveries of the target analytes with methanol were significantly higher than those achieved with the other two extraction solvents; hence, methanol was chosen for subsequent analyses. To obtain a good extraction recovery and absence of a significant matrix effect, 5F-MDMB-PICA and its metabolites were filtered with different filter membranes. This showed that the extraction recovery rates of 5F-MDMB-PICA and its metabolites on polyethersulfone (13 × 0.22 mm), nylon (13 × 0.22 mm), nylon (13 × 0.45 mm) and acrodisc (13 × 0.2 mm) filter membranes were high; however, because of their relative matrix effects, these filters were not selected. In addition, the material of polytetrafluoroethylene 1 (13 × 0.22 mm) and polytetrafluoroethylene 2 (13 × 0.22 mm) are the same, but the manufacturers of the two are different, which may cause different production processes. After investigating these two types of membranes, it was found that the extraction recoveries of polytetrafluoroethylene 1 (13 × 0.22 mm) and polytetrafluoroethylene 2 (13 × 0.22 mm) were essentially the same; both of them were higher than 85%, although the matrix effect of polytetrafluoroethylene 2 (13 × 0.22 mm) was better than that of polytetrafluoroethylene 1 (13 × 0.22 mm). Therefore, polytetrafluoroethylene 1 (13 × 0.22 mm) was chosen as the membrane for filtration.

### Method Validation

No interference was observed with any of the analytes, including the IS, in the 10 hair samples obtained from drug-free volunteers.

**Scheme 2:**
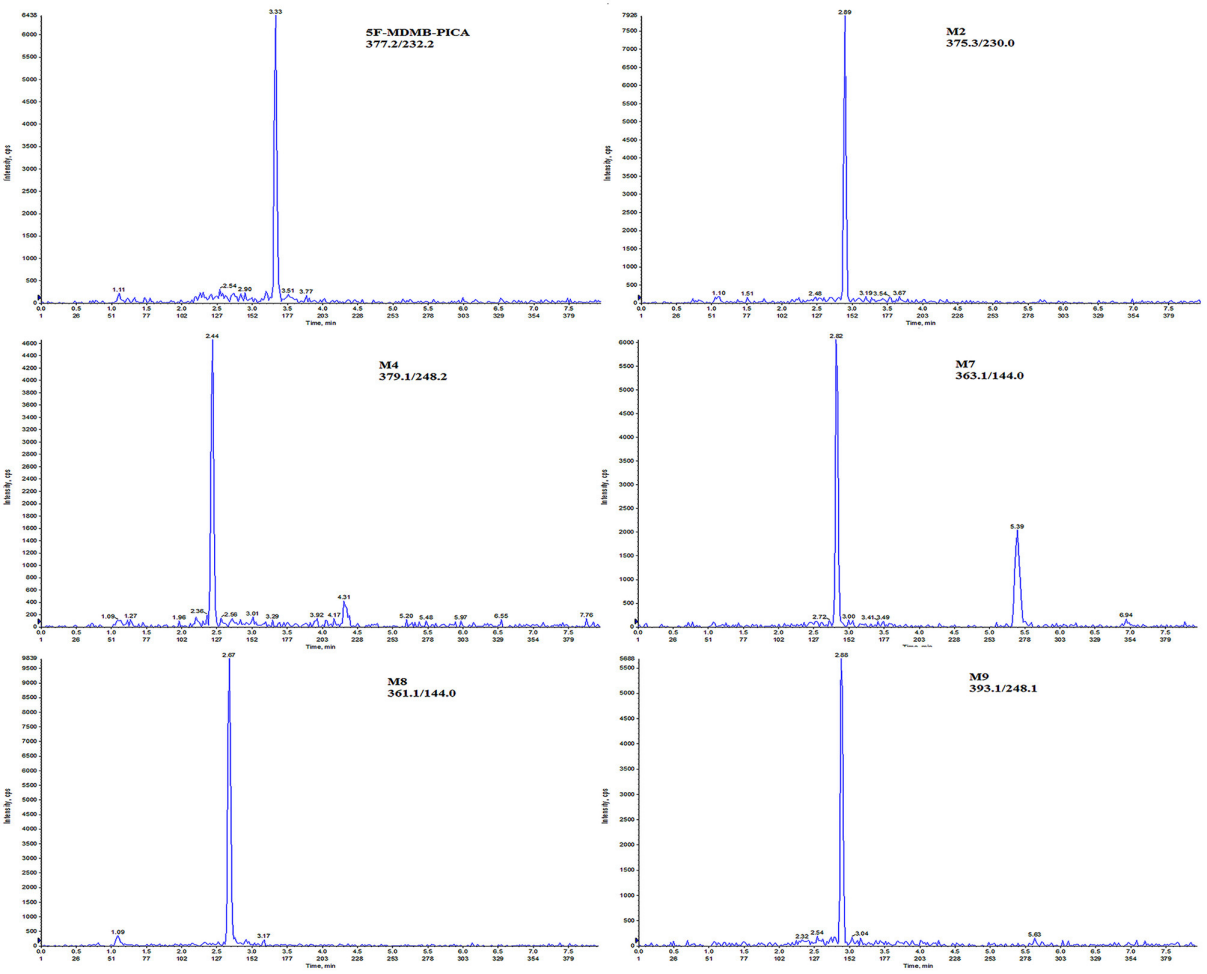
Chromatograms of 5F-MDMB-PICA and its metabolites extracted from human hair at the LLOQ (I pg/mg for 5F-MDMB-PICA, M2, M9; 2 pg/mg for M4, M7; and 5 pg/mg for M8).

The LOD and LLOQ of the analytes are shown in Table 1. The LOD ranged from 0.5 to 5 pg/mg, while the LLOQ ranged from 1 to 5 pg/mg (Scheme 2). According to the reference (Peters et al., 2007), for LLOQ, S/N is usually required to be equal to or >10; in contrast to the LLOQ determination, for LOD a S/N or k-factor equal to or greater than three is usually chosen. In our experiment, the S/N of 5F-MDMB-PICA, M2, M4, M7, M8, and M9 at LOD concentration are 3.1, 5.7, 3.8, 4.9, 5.6, and 3.6, respectively; while the S/N at LLOQ concentration are 10.8, 13.4, 11.2, 13.6, 12.7, and 10.9, respectively, and the precision and accuracy of LLOQ concentration meet criteria. In general, LLOQ concentration is 3 times the LOD concentration, but in some cases LLOQ concentration is not 3 times the LOD concentration. For example, according to the reference (Scientific Working Group for Forensic Toxicology, 2013), it is determined that the LOD concentration is 8.8 ng/mL and the LLOQ concentration is 10 ng/mL. Considering the reference literature and the experimental results of multiple compounds, it is determined that LLOQ concentration is twice the LOD concentration. This method had higher selectivity than those of other procedures published in the literature and exhibited improved sensitivity (Salomone et al., 2014). The linearity of each analyte is shown in Table 2, with correlation coefficients (*R*^2^) all higher than 0.999. The linear range of 5F-MDMB-PICA, M2 and M9 was 1–200 pg/mg; that of M4 and M7 was 2–200 pg/mg; and that of M8 was 5–200 pg/mg. The deviation value of the LLOQ concentration point on the linearity is <20%, while the deviation value of the other concentration points is <15%.

**Table 2:** Linearity, LOD, and LLOQ for 5F-MDMB-PICA and its metabolites in hair.

**Analytes**	**Linearity range (pg/mg) (*n* = 2)**	**Regression equations**	**Correlation coefficients (*R*^**2**^)**	**LOD (pg/mg)**	**LLOQ (pg/mg)**
5F-MDMB-PICA	1–200	y = 0.07073x + 0.01786	0.9992	0.5	1
M2	1–200	y = 0.08282x + 0.01135	0.9991	0.5	1
M4	2–200	y = 0.03617x – 0.00555	0.9994	1	2
M7	2–100	y = 0.04442x – 0.03089	0.9992	1	2
M8	5–200	y = 0.03365x – 0.00790	0.9992	5	5
M9	1–200	y = 0.08553x – 0.02541	0.9989	0.5	1

The intra- and inter-day precision and accuracy of all of the analytes at four levels are summarized in Table 3. The intra-day precision ranged from 0.7 to 10.8%, and the accuracy varied between 95.4 and 107.4% (*n* = 6). The inter-day precision ranged from 1.7 to 12.2%, and the accuracy varied between 95.0 and 102.8% (*n* = 6). In general, the RSD values of the intra- and inter-day precision and accuracy of the three QC samples were <15%, while the RSD values of the LLOQ samples were <20%. Intra-day and inter-day precision and accuracy values met the acceptance criteria.

**Table 3:** Intra-day and inter-day accuracy and precision, matrix effect and recovery for 5F-MDMB-PICA and its metabolites in hair.

**Analytes**	**Concentrations (pg/mg)**	**Intra-day (*****n*** **=** **6)**		**Inter-day (*****n*** **=24)**		**Matrix effect (*****n*** **=** **6)**		**Recovery (%) (*n* = 6)**
		**Precision (%)**	**Accuracy (%)**	**Precision (%)**	**Accuracy (%)**	**Mean (%)**	**CV (%)**	
5F-MDMB-PICA	1	6.6	105.0	5.8	98.8	23.0	1.2	72.5
	10	1.2	102.6	2.5	100.9	19.7	0.8	78.1
	50	4.0	99.8	4.9	101.3	19.1	1.7	91.8
	150	2.8	99.4	5.6	96.8	20.2	1.1	93.3
M2	1	1.2	97.4	8.3	95.0	53.5	4.9	83.8
	10	4.3	104.2	6.5	101.3	43.0	2.5	80.8
	50	4.5	99.0	7.4	101.0	40.6	4.1	81.5
	150	6.1	100.1	10.0	95.4	41.0	2.8	77.2
M4	2	1.2	101.2	5.0	102.3	90.4	11.4	64.4
	10	4.2	103.7	4.2	97.6	102.6	6.9	60.7
	50	4.1	98.0	4.5	99.6	98.3	6.6	76.0
	150	2.4	99.9	7.3	98.7	93.9	7.7	81.5
M7	2	0.7	104.2	7.8	105.7	55.6	4.3	63.3
	10	1.6	100.4	6.0	97.0	59.7	6.1	61.1
	50	1.8	96.1	4.6	99.8	59.2	7.2	77.5
	150	6.0	101.3	8.5	98.3	58.9	2.5	83.3
M8	5	2.8	97.7	5.3	101.3	30.2	2.2	63.0
	20	1.2	101.8	1.7	99.5	33.6	2.0	76.1
	50	1.3	97.6	4.1	98.3	35.6	4.2	79.4
	150	5.0	100.5	7.7	100.1	36.8	2.0	85.6
M9	1	10.8	107.4	12.2	101.0	57.2	8.3	70.6
	10	1.1	98.8	6.5	101.2	46.2	3.6	71.6
	50	1.5	95.4	6.9	98.6	46.0	2.6	70.8
	150	2.4	101.7	11.5	94.7	48.4	4.0	72.2

The results of the recovery and matrix effect are shown in Table 3. The matrix effect ranged from 19.1 to 102.6%. However, ion suppression occurred for 5F-MDMB-PICA and M8 in the hair matrix. Even at a low concentration, the matrix effect ranged from 19.7 to 33.6%. Whether this was indeed a systematic effect needs to be clarified; however, the relative standard deviation of the six different samples for each matrix was <5.8%. The recoveries of all the analytes were within a range of 61.1–93.3% for the three QC samples and for the LLOQ samples. According to the reference (Goebel et al., 2013), in hair matrix, however, only ion suppression was found. Even at a low concentration (5 pg/mg) absolute matrix values range from 15.4 to 71.5%. The matrix effect of compounds is easily affected by the concentration, and the lower the concentration, the higher the influence of the matrix effect. Affected by the physical and chemical properties of the compound, the polarity of 5F-MDMB-PICA is small, while the polarity of its metabolites is relatively large. We speculated that a competitive relationship was formed during the ionization process of 5F-MDMB-PICA and its metabolites. The ionization efficiency of 5F-MDMB-PICA was reduced, resulting in ion suppression. In addition, 5F-MDMB-PICA and its metabolites should be considered at the same time during sample preparation, which may also affect the matrix effect of 5F-MDMB-PICA. In the method validation, the linearity, precision and accuracy all meet the requirements.

The effect of dilution on precision and accuracy was investigated. The accuracies were 93–109%, and the RSD values were within 3.7%, demonstrating no detrimental impact of dilution.

The RSD values of 5F-MDMB-PICA and its metabolites stored at 4°C for 24, 48, and 72 h were 97–104%, 97–103%, and 97–105%, respectively. The RSD values were all within 20%, indicating that these analytes were sufficiently stable in hair. The stability results are shown in Table 4.

**Table 4:** Stability of 5F-MDMB-PICA and its metabolites in human hair.

**Analytes**	**Concentration (pg/mg)**	**Autosampler, 4**^****°****^**C**, ***n*** **=** **6 (%)**		
		**24 h**	**48 h**	**72 h**
5F-MDMB-PICA	1	100.6	100.8	105.0
	10	100.2	99.2	100.8
	50	99.7	100.4	99.1
	150	100.0	99.8	101.0
M2	1	102.2	103.4	103.7
	10	98.9	101.0	100.5
	50	97.4	98.2	99.7
	150	101.2	100.6	100.5
M4	2	103.7	101.8	96.8
	10	99.2	100.0	100.9
	50	94.7	96.7	99.6
	150	101.8	101.0	99.1
M7	2	101.7	104.4	105.3
	10	99.4	98.7	101.6
	50	99.7	96.8	99.8
	150	100.2	100.8	100.6
M8	5	100.6	103.4	102.5
	20	100.4	100.8	104.4
	50	98.7	95.0	94.3
	150	100.4	101.0	101.4
M9	1	97.1	105.8	102.2
	10	103.9	100.3	100.2
	50	100.2	98.0	97.0
	150	99.6	100.9	101.3

### Application to Cases

**Scheme 3:**
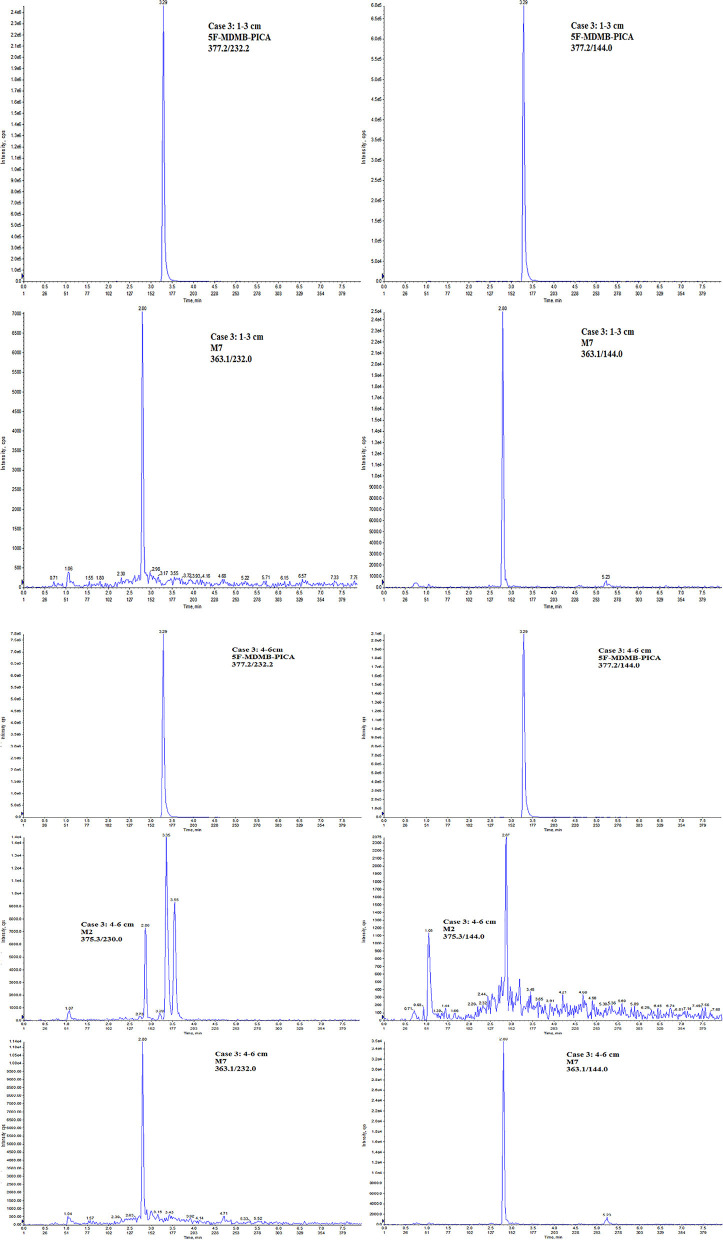
Ion chromatograms of case 3.

The LC-MS/MS method was applied to the determination of 5F-MDMB-PICA and its metabolites in four authentic forensic cases (see Table 5 and Scheme 3).

**Table 5:** Case Information and toxicology results.

**Case**	**Age**	**Sex**	**Hair section (cm)**	**Concentration (pg/mg)**				
				**5F-MDMB-PICA**	**M2**	**M4**	**M7**	**M8**	**M9**
1	22	Female	1–3	77	nd	nd	<2^*^	nd	nd
			4–6	283	<1^*^	nd	<2^*^	nd	nd
2	53	Male	1–3	46	nd	<2^*^	2.7	<5^*^	<1^*^
			4–6	349	<1^*^	<2^*^	<2^*^	<5^*^	<1^*^
3	25	Male	1–3	275	<1^*^	nd	2.8	<5^*^	nd
			4–6	1025	3.0	<2^*^	5.6	<5^*^	<1^*^
4	36	Male	1–3	2	nd	<2^*^	<2^*^	<5^*^	<1^*^
			4–6	nd	nd	nd	nd	nd	nd

* *detected below the LLOQ; nd, not detected*.

#### Case 1

A 23-year-old female with a history of smoking was introduced by a friend that smoking e-liquid is more fun than smoking regular tobacco cigarettes. She smoked e-liquids many times without knowing their composition. The individual developed hallucinations and euphoria after smoking and fell down a set of stairs. She was found by her family, who promptly sent her to the hospital. She recovered after rescue and 5F-MDMB-PICA and its metabolites were detected in her hair. The concentrations of 5F-MDMB-PICA were 77 and 283 pg/mg in the proximal 1–3 and 4–6 cm sections of her hair, respectively. Only metabolite M7 was detected in the 1–3 cm sections, and its concentration was lower than the LLOQ. The metabolites M2 and M7 were detected at 4–6 cm, and their concentrations were also lower than the LLOQ.

#### Case 2

A 53-year-old male with high blood pressure purchased e-liquids several times on the Internet. After the last inhalation, he developed tachycardia, convulsions and shock. Upon discovery, he was admitted to the hospital for treatment but died of cerebral hemorrhage. 5F-MDMB-PICA and its metabolites were detected in both 1–3 and 4–6 cm sections of the hair. Metabolites M4, M7, M8, and M9 were detected in the 1–3 cm hair section, and their concentrations were all lower than the LLOQ. All metabolites are detected in the 4–6 cm hair section and were lower than the LLOQ.

#### Case 3

A 25-year-old male with a long history of drug abuse attended a gathering of friends. He drank alcohol and smoked e-liquid and K powder at the party. During this period, he suddenly experienced shock, convulsions and tachycardia. This individual was seen by a doctor soon after and recovered without sequelae. The police found a large amount of e-liquid and K powder at the scene. According to the existing method of our forensic toxicology laboratory, the concentration of K powder in the hair was qualitatively and quantitatively determined to be 200 pg/mg (Zhuo et al., 2020). The concentrations of 5F-MDMB-PICA detected in the 1–3 and 4–6 cm hair sections were 275 and 1,025 pg/mg, respectively. The concentration of metabolite M7 was 2.8 pg/mg in the 1–3 cm hair section, and the concentrations of M2 and M8 were both lower than the LLOQ. In addition, the concentrations of metabolites M2 and M7 were detected in the 4–6 cm hair section at 3.0 and 5.6 pg/mg, respectively, and the concentrations of the remaining metabolites were lower than the LLOQ.

#### Case 4

A 36-year-old male was meeting with friends at an entertainment venue. The police received a report that someone in the crowd took drugs. A man and his friend were detained and found to have a large amount of suspicious white powder and liquid at the scene. The man said that he had smoked the e-liquid for the first time under unknown circumstances. After testing, the suspicious white powder and liquid at the scene were found to be fentanyl and 5F-MDMB-PICA. No fentanyl was detected in his hair (Qin et al., 2019). The 5F-MDMB-PICA concentration was 2 pg/mg, and its metabolites were lower than the LLOQ in the 1–3 cm hair section of the individual, while 5F-MDMB-PICA and its metabolites were negative in the 4–6 cm sections.

### Comparison With Previous Research

It has been reported in some studies that LC-MS/MS methods can be used to analyse other synthetic cannabinoids in human hair. The limit of detection of this study was the same as that of a previous study and reached the pg level (Arbouche et al., 2019; Cho et al., 2020). Chinese hair is similar to Korean and Japanese hair. Cho et al. (2020) studied the distribution of synthetic cannabinoids and metabolites in the hair of Korean drug users. The concentrations of AB-CHMINCA and its metabolite M2 were 2.5–13,500.0 and 0.5–35.1 pg/mg, respectively, and the concentration of the precursor drug was greater than the concentrations of metabolites in all cases. Moreover, Arbouche et al. (2019) detected a higher concentration of AB-PINACA in head hair than in pubic hair and detected a very low concentration of AB-PINACA in urine, which may be caused by rapid metabolism, and pubic hair is easily contaminated by urine.

The growth rate of human hair is ~0.7–1.4 cm per month, and hair can accurately reflect the medication history of an individual. Generally, a proximal 1 cm of hair can roughly reflect the medication history of an individual in the past month. We analyzed 5F-MDMB-PICA and its metabolites in the 1–6 cm section in four individuals suspected of drug use, reflecting the medication situation in the past 6 months. The individuals in cases 1 and 2 had 5F-MDMB-PICA in their 1–6 cm hair section samples, and the metabolite concentrations were low, below the LLOQ, indicating that the drug had been consumed within 6 months, but only a small amount of the drug was consumed. The individual from case 3 had higher concentrations of the precursor drug and its metabolites than the individuals from cases 1 and 2 in the 1–6 cm hair section, indicating that he had taken 5F-MDMB-PICA for a long time and had consumed a large amount of this drug in the past 6 months. In case 4, 5F-MDMB-PICA was only detected in the 1–3 cm hair section of the individual, the concentration was very low, and the concentrations of metabolites were lower than the LLOQ, supporting the idea that the individual had taken the drug for the first time. The concentration of 5F-MDMB-PICA detected in human hair samples is much higher than the concentration of its metabolites. 5F-MDMB-PICA undergoes metabolic reactions such as hydrolysis, hydroxylation or oxidation in the body to produce metabolites, which increase the polarity of metabolites (Mogler et al., 2018). Therefore, metabolites are not easily combined with the keratin matrix in human hair, which may result in lower levels of metabolites in human hair. In addition, studies have reported that some people have detected synthetic cannabinoids in hair samples of individuals that have not taken those drugs, which indicates that some drug powders or fumes can cause external contamination of hair samples or that an additional route of synthetic cannabinoid absorption by hair, such as sidestream smoke, may cause false positive results (Hutter et al., 2012; Saito et al., 2014). Therefore, analysis of the precursor drug and its metabolites in hair provides useful information for analysis. In this study, we detected metabolites of 5F-MDMB-PICA when analyzing 4 real hair samples. Metabolite M7 (*m/z* 363.1) is the most abundant metabolite among the 4 real hair samples. According to previously reported data on SCs containing tert-leucine methyl ester substituents (such as MDMB-CHMICA), this metabolic step of ester hydrolysis is usually one of the main reactions *in vivo* (Grigoryev et al., 2016). In addition, we detected four other metabolites of 5F-MDMB-PICA in real human hair samples, M2, M4, M8, and M9. Previously, Mogler et al. (2018) detected 12 phase I metabolites of 5F-MDMB-PICA in the analysis of real urine samples. Compound M12 (*m/z* 363), which is the same as metabolite M7 in human hair, is also the most abundant metabolite in all real urine samples and is formed by the hydrolysis of terminal methyl esters. As described in Table 4, it was found that 5F-MDMB-PICA was the main compound detected in the hair samples, and its abundance was higher than that those of its metabolites M2, M4, M7, M8, and M9. The metabolites in this study are the same as the metabolites of 5F-MDMB-PICA detected in urine (Mogler et al., 2018). In addition, metabolite M7 is also formed as an artificial product through the hydrolysis of methyl esters during smoking. If M7 condenses on hair, it may be falsely determined as 5F-MDMB-PICA consumption when analyzing the metabolites of synthetic cannabinoids in hair. Therefore, the interpretation of metabolites should be treated with caution.

## Conclusion

This study describes the development and validation of an LC-MS/MS procedure for the identification and quantification of 5F-MDMB-PICA and its metabolites in human hair. The method was successfully applied to four individuals suspected of the use of a synthetic cannabinoid in which 5F-MDMB-PICA was detected.

## Data Availability Statement

The raw data supporting the conclusions of this article will be made available by the authors, without undue reservation.

## Author Contributions

LZ wrote the article and completed the experiment. YS revised the article and guided the experiment. HW and PX provided fund support and guidance for the experiment. WL and OD provided guidance for the experiment and instruments. HC, BS, and MS provided real materials. LL, ML, and HQ provided help for the experiment.

## Conflict of Interest

The authors declare that the research was conducted in the absence of any commercial or financial relationships that could be construed as a potential conflict of interest.
